# Enhancement of porcine in vitro embryonic development through luteolin-mediated activation of the Nrf2/Keap1 signaling pathway

**DOI:** 10.1186/s40104-023-00947-9

**Published:** 2023-12-01

**Authors:** Se-Been Jeon, Pil-Soo Jeong, Min Ju Kim, Hyo-Gu Kang, Bong-Seok Song, Sun-Uk Kim, Seong-Keun Cho, Bo-Woong Sim

**Affiliations:** 1 Futuristic Animal Resource & Research Center (FARRC), Korea Research Institute of Bioscience and Biotechnology (KRIBB), Cheongju, 28116 Republic of Korea; 2 Department of Animal Science, College of Natural Resources & Life Science, Pusan National University, Miryang, 50463 Republic of Korea; 3 Department of Animal Science and Biotechnology, College of Agriculture and Life Science, Chungnam National University, Daejeon, 34134 Republic of Korea; 4 Department of Functional Genomics, University of Science and Technology, Daejeon, 34113 Republic of Korea; 5 Department of Animal Science, Life and Industry Convergence Research Institute (RICRI), College of Natural Resources & Life Science, Pusan National University, Miryang, 50463 Republic of Korea

**Keywords:** Luteolin, Mitochondrial function, Nrf2/Keap1 signaling pathway, Oxidative stress, Porcine embryo development

## Abstract

**Background:**

Oxidative stress, caused by an imbalance in the production and elimination of intracellular reactive oxygen species (ROS), has been recognized for its detrimental effects on mammalian embryonic development. Luteolin (Lut) has been documented for its protective effects against oxidative stress in various studies. However, its specific role in embryonic development remains unexplored. This study aims to investigate the influence of Lut on porcine embryonic development and to elucidate the underlying mechanism.

**Results:**

After undergoing parthenogenetic activation (PA) or in vitro fertilization, embryos supplemented with 0.5 µmol/L Lut displayed a significant enhancement in cleavage and blastocyst formation rates, with an increase in total cell numbers and a decrease in the apoptosis rate compared to the control. Measurements on D2 and D6 revealed that embryos with Lut supplementation had lower ROS levels and higher glutathione levels compared to the control. Moreover, Lut supplementation significantly augmented mitochondrial content and membrane potential. Intriguingly, activation of the Nrf2/Keap1 signaling pathway was observed in embryos supplemented with Lut, leading to the upregulation of antioxidant-related gene transcription levels. To further validate the relationship between the Nrf2/Keap1 signaling pathway and effects of Lut in porcine embryonic development, we cultured PA embryos in a medium supplemented with brusatol, with or without the inclusion of Lut. The positive effects of Lut on developmental competence were negated by brusatol treatment.

**Conclusions:**

Our findings indicate that Lut-mediated activation of the Nrf2/Keap1 signaling pathway contributes to the enhanced production of porcine embryos with high developmental competence, and offers insight into the mechanisms regulating early embryonic development.

**Supplementary Information:**

The online version contains supplementary material available at 10.1186/s40104-023-00947-9.

## Background

In vitro production (IVP) of embryos, which encompasses in vitro culture (IVC), serves as the cornerstone for various embryo engineering techniques. It is an invaluable asset for animal husbandry, assisted reproductive technologies, and the study of early embryogenesis mechanisms [1]. Although numerous studies have sought to refine IVC conditions to enhance the quality of IVP embryos, the in vivo microenvironment during embryonic development remains challenging to replicate fully [2, 3]. Consequently, there is an urgent need for the continual development and optimization of IVC systems to improve IVP embryo quality.

One of the primary challenges facing IVP embryos is their reduced developmental capacity. This is attributed to the oxidative stress induced by exposure to light, oxygen, and oxidizing agents during IVP, which leads to an increase in reactive oxygen species (ROS) levels [4]. The ROS family, which includes the superoxide radical, hydrogen peroxide, and hydroxyl radical, is produced as a byproduct of intracellular metabolic activities, particularly in the mitochondrial respiratory chain. ROS are critical in regulating various cellular signaling pathways, including proliferation, differentiation, and survival [5]. However, excessive intracellular accumulation of ROS disrupts redox homeostasis, leading to oxidative stress. This can have a range of detrimental effects, including lipid peroxidation, protein denaturation, DNA damage, mitochondrial dysfunction, and apoptosis [6, 7]. In the in vivo environment, the female reproductive tract synthesizes several antioxidant enzymes, such as catalase (CAT), superoxide dismutase (SOD), and glutathione peroxidase (GPX). These enzymes are part of antioxidant systems that protect against ROS-induced damage. However, these systems are often deficient in IVC conditions [8]. This is particularly concerning for porcine embryos, which are highly susceptible to changes in the culture environment due to their elevated cellular lipid content compared to other species [9]. Previous studies have attempted to mitigate oxidative stress by supplementing IVC media with various ROS scavengers, such as vitamin C, melatonin, and lycopene [10–12]. However, the efficiency of porcine IVP still falls short when compared to the natural in vivo environment. This highlights the need to identify more potent antioxidants and to understand the underlying mechanisms governing the developmental capacity of porcine IVP embryos.

Luteolin (Lut), a flavonoid compound, also recognized as 3,4,5,7-tetrahydroxyflavone, is present in a diverse array of plants, including herbs, vegetables, and fruits [13]. Lut possesses an assortment of pharmacological properties owing to its ability to modulate redox states. These properties encompass anti-inflammatory, antibacterial, anticancer, antiangiogenic, and neuroprotective activities [14]. Importantly, Lut possesses antioxidant activity and has been shown to curtail the formation of free radicals, thereby combating oxidative stress in numerous cell types [15–17]. While the biological and physiological functions of Lut have been extensively documented, its impact on porcine in vitro embryonic development and the associated signaling pathways remains unexplored.

The nuclear factor erythroid-2-related factor 2 (Nrf2)/Kelch-like ECH-associated protein 1 (Keap1) signaling pathway is widely recognized as a key oxidative stress defense mechanism, as it regulates the expression of antioxidant enzymes and genes related to redox homeostasis [18]. Under normal conditions, Nrf2 is ubiquitinated by Keap1 in the cytoplasm and subsequently degraded through the proteasome pathway. However, when cells experience electrophilic or oxidative stress, Nrf2 dissociates from Keap1 and migrates to the nucleus. There, it binds to the antioxidant response element (ARE) and orchestrates antioxidant activity by modulating the expression of genes involved in detoxification and antioxidant defense mechanisms [19]. Among the target genes are *SOD*, *CAT*, *GPX*, heme oxygenase-1, and NAD(P)H quinone oxidoreductase 1, which are critical for cellular survival and are instrumental in neutralizing harmful oxidants within the cellular environment [20]. Therefore, the induction of this signaling pathway using external activators may hold significant promise for managing excessive ROS accumulation and fostering embryonic development.

Numerous studies have highlighted the protective role of Lut against oxidative stress through the activation of the Nrf2/Keap1 signaling pathway [21, 22]. However, evidence elucidating the relationship between Lut and the Nrf2/Keap1 signaling pathway specifically in porcine in vitro embryonic development is currently lacking. Consequently, this study aimed to investigate the porcine in vitro embryonic development and to elucidate the underlying mechanism by adding optimal concentrations of Lut to the IVC medium. This was achieved by assessing the quality of the blastocysts, monitoring intracellular levels of ROS and glutathione (GSH), and evaluating mitochondrial content and membrane potential. Additionally, we sought to discern the connection between the role of Lut and the Nrf2/Keap1 signaling pathway during porcine early embryogenesis.

## Methods

### Chemicals

All the chemicals and reagents employed in this study were procured from Sigma-Aldrich Chemical Company (St. Louis, MO, USA), except where specified otherwise.

### Oocyte collection and in vitro maturation (IVM)

Porcine ovaries were sourced from a local abattoir in close proximity. The ovaries were transported in 0.9% saline solution, supplemented with 75 μg/mL of benzyl-penicillin potassium G and 50 μg/mL of streptomycin sulfate, at a temperature of 38.5 °C. Cumulus-oocyte complexes (COCs) were extracted from follicles of 3 to 6 mm in diameter by aspiration, using a disposable 10-mL syringe fitted with an 18-gauge needle. The COCs were then rinsed in 0.9% saline solution enriched with 0.1% bovine serum albumin (BSA). Subsequently, the COCs underwent maturation in IVM medium (Tissue Culture Medium 199, complemented with 10% porcine follicular fluid, 0.57 mmol/L cysteine, 10 ng/mL epidermal growth factor, and 25 μmol/L β-mercaptoethanol), with the addition of 10 IU/mL pregnant mare serum gonadotropin (HOR-272, Prospec, Israel) and 10 IU/mL Human Chorionic Gonadotropin (HOR-250, Prospec). This maturation process was carried out in a 4-well dish (Nunc, Roskilde, Denmark) under an atmosphere of 5% CO_2_ at 38.5 °C for an initial period of 22 h. Following the initial 22 h maturation period, the COCs underwent further maturation in IVM medium, this time without hormones, for an additional 22 h. Post-IVM, the cumulus cells were detached by gentle pipetting in the presence of 0.1% hyaluronidase. Only oocytes displaying a visible polar body, indicative of normal morphology and homogeneous cytoplasm, were used in subsequent experiments.

### Parthenogenetic activation (PA), in vitro fertilization (IVF), and IVC

For PA, selected metaphase II oocytes were exposed to 15 μmol/L of ionomycin in the dark for 5 min in Dulbecco’s phosphate-buffered saline (DPBS; Gibco, Carlsbad, CA, USA) supplemented with 60 μg/mL gentamicin sulfate salt, 75 μg/mL streptomycin sulfate, and 4 mg/mL BSA. Subsequently, these metaphase II oocytes were cultured in IVC medium (porcine zygote medium-3 containing 4 mg/mL BSA) supplemented with 5 µg/mL cytochalasin B and 2 mmol/L 6-dimethylaminopurine under a 5% CO_2_ atmosphere at 38.5 °C for 4 h. After this period, the activated oocytes were transferred to fresh IVC medium and incubated under 5% CO_2_ at 38.5 °C for 6 d.

For IVF, the modified Tris-buffered medium (mTBM) was prepared with 113.1 mmol/L NaCl, 3 mmol/L KCl, 7.5 mmol/L CaCl_2_·2H_2_O, 20 mmol/L Tris (Fisher Scientific, Waltham, MA, United States), 11 mmol/L glucose, 5 mmol/L sodium pyruvate, 2.5 mmol/L caffeine sodium benzoate, and 1 mg/mL BSA. Fresh swine spermatozoa were washed three times with sperm washing medium (DPBS containing 60 μg/mL gentamicin sulfate salt, 75 μg/mL streptomycin sulfate, and 1 mg/mL BSA). After washing, 2 mL of sperm washing medium was gently added to the spermatozoa pellet, followed by incubation for 15 min under 5% CO_2_ at 38.5 °C. The spermatozoa were then resuspended in 1 mL of mTBM. A 2-μL aliquot of this diluted spermatozoa solution was added to mTBM containing 10–15 oocytes, achieving a final concentration of 1.5 × 10^5^ spermatozoa/mL. Oocytes and spermatozoa were co-incubated under 5% CO_2_ at 38.5 °C for 6 h. Following this, attached spermatozoa were removed by gentle pipetting, and the embryos were cultured in IVC medium under 5% CO_2_ at 38.5 °C for 6 d. Cleavage and blastocyst formation were evaluated on 2 and 6 d, respectively.

### Chemical treatment

Lut (L9283, purity ≥ 98%; Sigma-Aldrich) was dissolved in dimethyl sulfoxide (DMSO) and further diluted with IVC medium to achieve final concentrations of 0, 0.05, 0.5, and 5 μmol/L. To investigate the correlation between Lut and the Nrf2/Keap1 signaling pathway, brusatol (SML1868; Sigma-Aldrich) was also dissolved in DMSO and diluted with IVC medium to reach final concentrations of 0, 20, 50, and 100 nmol/L. The final DMSO concentration in the IVC medium was maintained below 0.2%.

### Terminal deoxynucleotidyl transferase-mediated dUTP-digoxygenin nick end-labeling (TUNEL) assay

The TUNEL assay was performed using the In Situ Cell Death Detection Kit (Roche, Basel, Switzerland). Blastocysts were fixed in a formalin solution overnight. After fixation, the blastocysts were washed three times in DPBS containing 0.1% polyvinyl alcohol (PVA-PBS) and then permeabilized in DPBS containing 1% (v/v) Triton X-100 at room temperature (RT) for 1 h. Following this, blastocysts were washed three times with PVA-PBS and stained with fluorescein-conjugated dUTP and terminal deoxynucleotidyl transferase for 1 h at 38.5 °C. After incubation, the blastocysts were rinsed three times in PVA-PBS and mounted on slides with Vectashield containing DAPI (Vector Laboratories, Burlingame, CA, USA). DAPI-labeled and TUNEL-positive nuclei were visualized using a fluorescence microscope (DMi8; Leica, Wetzlar, Germany).

### Measurement of intracellular ROS and GSH Levels

The intracellular levels of ROS and GSH were measured using CM-H2DCFDA (Invitrogen, Carlsbad, CA, USA) and CMF2HC (Invitrogen), respectively. Embryos on D2 and blastocysts on D6 were washed three times with PVA-PBS and then incubated in PVA-PBS containing 5 μmol/L CM-H2DCFDA (for ROS) or 10 μmol/L CMF2HC (for GSH) for 10 min and 30 min, respectively. After incubation, both the D2 embryos and D6 blastocysts were washed three times with PVA-PBS, and their fluorescence was observed under a fluorescence microscope (Leica) with ultraviolet filters (460 nm for ROS and 370 nm for GSH). Fluorescence intensities were analyzed using ImageJ software (version 1.47; National Institutes of Health, Bethesda, MD, USA) and normalized to the control embryo size.

### Assessment of mitochondrial content and membrane potential

The content of active mitochondria and the mitochondrial membrane potential were evaluated using MitoTracker Red CMXRos (Invitrogen) and tetramethylrhodamine, methyl ester (TMRM; Invitrogen), respectively. For MitoTracker staining, D2 embryos and D6 blastocysts were washed three times with PVA-PBS and then incubated in PVA-PBS containing 200 nmol/L MitoTracker for 30 min. Following incubation, they were washed three times with PVA-PBS and observed under a fluorescence microscope (Leica). For TMRM staining, D2 embryos and D6 blastocysts underwent a similar process, but with incubation in PVA-PBS containing 200 nmol/L TMRM for 30 min. Subsequently, they were stained with 10 μg/mL Hoechst 33342 for 10 min and then observed under a fluorescence microscope (Leica). Fluorescence intensities were quantified using ImageJ software and normalized to the control embryo size.

### Immunocytochemistry

Blastocysts were fixed in a formalin solution overnight and subsequently washed three times in PVA-PBS. They were then permeabilized with DPBS containing 1% Triton X-100 at RT for 1 h. After permeabilization, blastocysts were washed three more times in PVA-PBS and transferred to a blocking solution (DPBS containing 0.05% v/v Tween 20 and 2 mg/mL BSA) for 1 h at RT. Following this, blastocysts were incubated with primary antibodies Nrf2 (1:200; ab31163; Abcam, Cambridge, MA, United States) and Keap1 (1:200; ab226997; Abcam) overnight at 4 °C [23]. After incubation with primary antibodies, blastocysts were washed three times with DPBS containing 0.05% v/v Tween 20 and again placed in blocking solution for 1 h at RT. They were then incubated with the secondary antibody (1:200; Alexa Fluor 488-labeled goat anti-rabbit) for 1 h at RT. Finally, the blastocysts were washed with DPBS containing 0.05% v/v Tween 20 and mounted on slides with DAPI. Signal intensities were observed under a fluorescence microscope (Leica), and fluorescence intensities were quantified using ImageJ software and normalized to the control embryo size.

### Quantitative real-time polymerase chain reaction

Poly(A) mRNAs were extracted from blastocysts using the Dynabeads mRNA Direct Kit (Invitrogen), according to the manufacturer's instructions. The samples were lysed for 5 min at RT using 100 μL of lysis/binding buffer, and 30 μL of Dynabeads oligo (dT)_25_ was added to each sample. Subsequently, the beads were separated from the binding buffer using a Dynal magnetic bar (Invitrogen). The bound poly(A) mRNA and beads were isolated by washing with washing buffers A and B, followed by the addition of 7 μL of Tris buffer. Reverse transcription was then carried out using the Prime Script RT Reagent Kit with gDNA Eraser (Takara Bio Inc., Shiga, Japan), as per the manufacturer's instructions. The resulting cDNA was used as a template for PCR amplification, with PCR conditions set at 95 °C for 5 min, followed by 40 cycles of 95 °C for 20 s and 60 °C for 20 s. The experiments were conducted using an Mx3000P QPCR system (Agilent, Santa Clara, CA, United States) and SYBR Premix Ex Taq (Takara Bio Inc.). The primers used in the study are listed in Additional file 1.

### Experimental design

We aimed to investigate the effect of Lut on porcine early embryogenesis. We conducted experiments using PA embryos to prevent variations due to sperm factors associated with IVF. In Exp. 1, a total of 783 PA embryos were used for five independent replicates to evaluate the effect of Lut at various concentrations (0, 0.05, 0.5, and 5 µmol/L) on cleavage and blastocyst formation rate, and total cell numbers. Based on embryonic development results, we chose a 0.5-µmol/L concentration of Lut for following experiments. A total of 84 PA blastocysts were used for three independent replicates to evaluate the cellular apoptosis and a total of 120 PA blastocysts were used for three independent replicates to examine the mRNA expression levels of developmental potential and apoptosis-related genes. In Exp. 2, we examined the antioxidant activity of Lut on porcine early embryogenesis. A total of 96 D2 4-cell embryos and 52 D6 blastocysts were used for three independent replicates to evaluate the intracellular ROS levels and a total of 72 D2 4-cell embryos and 48 D6 blastocysts were used for three independent replicates to evaluate the intracellular GSH levels. In Exp. 3, we examined the effect of Lut on mitochondrial functions in porcine PA embryos. A total of 102 D2 4-cell embryos and 52 D6 blastocysts were used for three independent replicates to evaluate the mitochondrial content and a total of 46 D2 4-cell embryos and 48 D6 blastocysts were used for three independent replicates to evaluate the mitochondrial membrane potential. In Exp. 4, we examined the expression levels of proteins and mRNAs associated with the Nrf2/Keap1 signaling pathway in porcine early embryogenesis. A total of 48 and 60 D6 blastocysts were used for three independent replicates to analyze the expression levels of Nrf2 and Keap1 by immunocytochemistry and a total of 120 D6 blastocysts were used for three independent replicates to analyze the mRNA expression levels of downstream target genes associated with the Nrf2/Keap1 signaling pathway. To elucidate the interplay between the Nrf2/Keap1 signaling pathway and the beneficial effects of Lut, we applied 50 nmol/L brusatol (an Nrf2 inhibitor) based on our preliminary experiment (Additional file 6: Table S6, Additional file 9: Fig. S1). In Exp. 5, a total of 541 PA embryos were used for five independent replicates to evaluate the effect of Lut and brusatol on cleavage and blastocyst formation rate. A total of 63 D6 PA blastocysts were used for five independent replicates to evaluate the cellular apoptosis. In Exp. 6, a total of 108 and 114 D2 4-cell embryos were used for three independent replicates to evaluate the effect of Lut and brusatol on intracellular ROS and GSH levels. A total of 126 and 72 D2 4-cell embryos were used for three independent replicates to evaluate the effect of Lut and brusatol on mitochondrial content and membrane potential.

### Statistical analyses

All experiments were conducted at least in triplicate, and data are presented as the mean ± standard error of the mean. A *P*-value less than 0.05 was considered statistically significant. Data for two groups were compared using Student's *t*-test, while data for three or more groups were analyzed by one-way ANOVA (Analysis of Variance), followed by the Tukey–Kramer test. We used SigmaStat software (version 18; SPSS Inc., Chicago, IL, USA) for all statistical analyses.

## Results

### Lut enhances the developmental competence of porcine IVP embryos

**Fig. 1:**
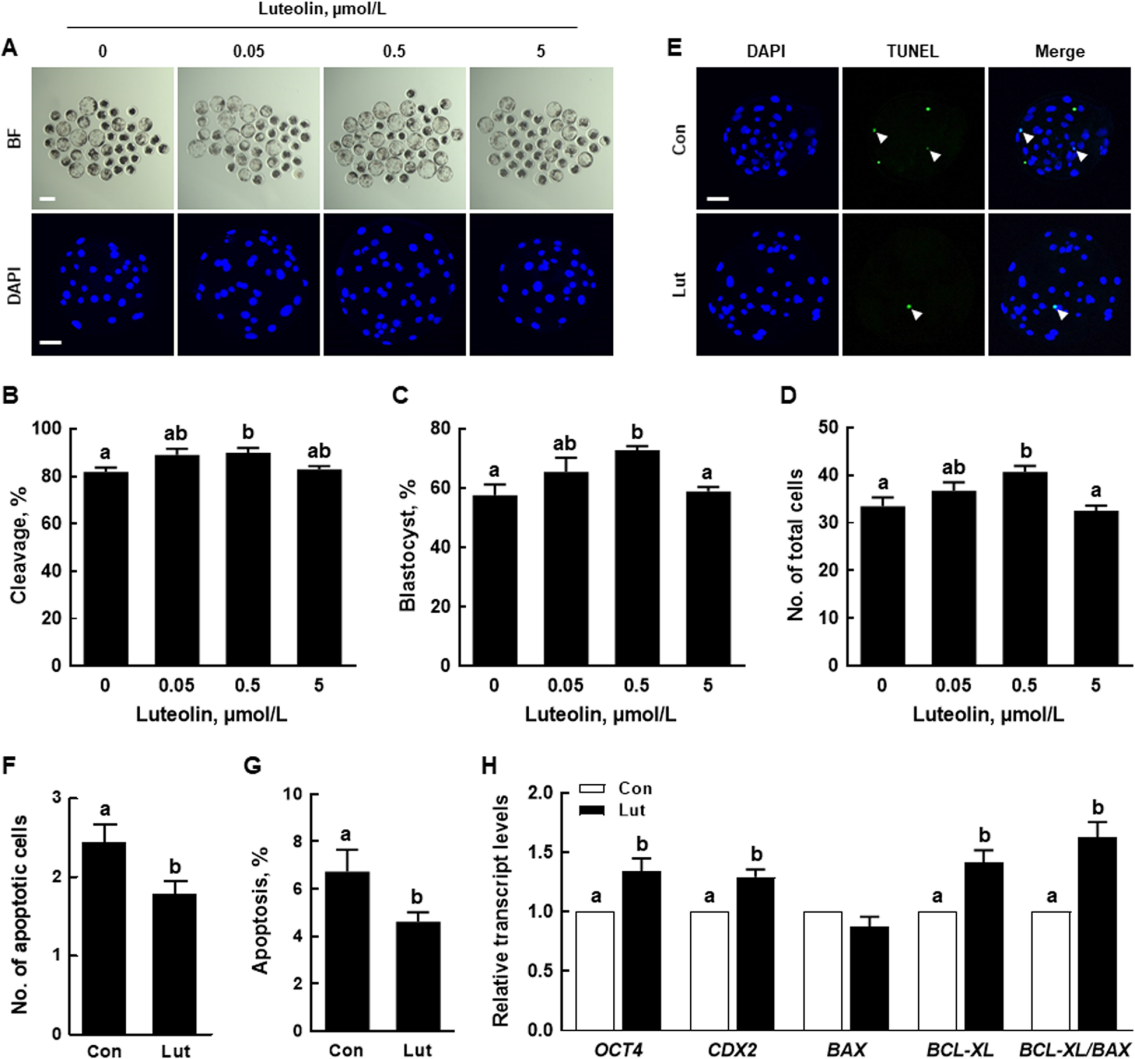
Effects of luteolin (Lut) on the developmental competence of porcine parthenogenetic activation (PA) embryos. **A** Representative bright-field images (upper, scale bar = 200 µm) and nuclear-stained images (lower, scale bar = 50 µm) of blastocysts cultured with or without varying concentrations of Lut. **B**-**D** Quantification of cleavage rate, blastocyst formation rate, and total cell number in the indicated groups (0; *n* = 197, 0.05; *n* = 196, 0.5; *n* = 195, 5; *n* = 195). **E** Terminal Deoxynucleotidyl Transferase-Mediated dUTP Nick End Labeling (TUNEL) assay of blastocysts in the indicated groups. Embryos were stained for TUNEL (green, indicated by white arrows) and nuclei (blue). Scale bar = 50 µm. **F**, **G** Quantification of the number and proportion of apoptotic cells in the indicated groups (*n* = 42 per group). **H** Quantitative RT-PCR results for developmental potential and apoptosis-related genes in blastocysts (*n* = 3 per group). The data are derived from at least three independent experiments, and means with similar superscripts do not differ (*P* > 0.05)

To explore the impact of Lut on porcine early embryogenesis, we cultured PA embryos in a culture medium supplemented with various concentrations of Lut (0, 0.05, 0.5, and 5 µmol/L) for 6 d. The cleavage rate, blastocyst formation rate, and total cell numbers significantly increased with the supplementation of 0.5 µmol/L Lut, in comparison to the control (Fig. 1A–D, Additional file 2: Table S2). Through the TUNEL assay, we found that 0.5 µmol/L Lut supplementation significantly reduced both the number of apoptotic cells and the apoptosis rate compared to the control (Fig. 1E–G, Additional file 3: Table S3). Additionally, the expression levels of developmental potential-related genes (*OCT4* and *CDX2*) were significantly elevated in the Lut group compared to the control. Although there was no significant difference in the expression levels of the pro-apoptosis-related gene (*BAX*), the expression levels of the anti-apoptosis-related gene (*BCL-XL*) and the *BCL-XL*/*BAX* ratio were significantly higher in the Lut group than in the control (Fig. 1H). Based on these findings, we selected 0.5 µmol/L Lut as the optimal concentration for promoting porcine embryonic development.

Furthermore, we investigated whether Lut supplementation could enhance the developmental competence of IVF embryos. In line with the results for PA, all parameters of developmental competence, including cleavage rate, blastocyst formation rate, total cell number, and cellular survival, were significantly improved with Lut supplementation compared to the control (Additional files 4 and 5: Tables S4 and S5, Additional file 9: Fig. S1). These results suggest that Lut supplementation positively affects porcine early embryogenesis.

### Lut reduces oxidative stress in porcine PA embryos

**Fig. 2:**
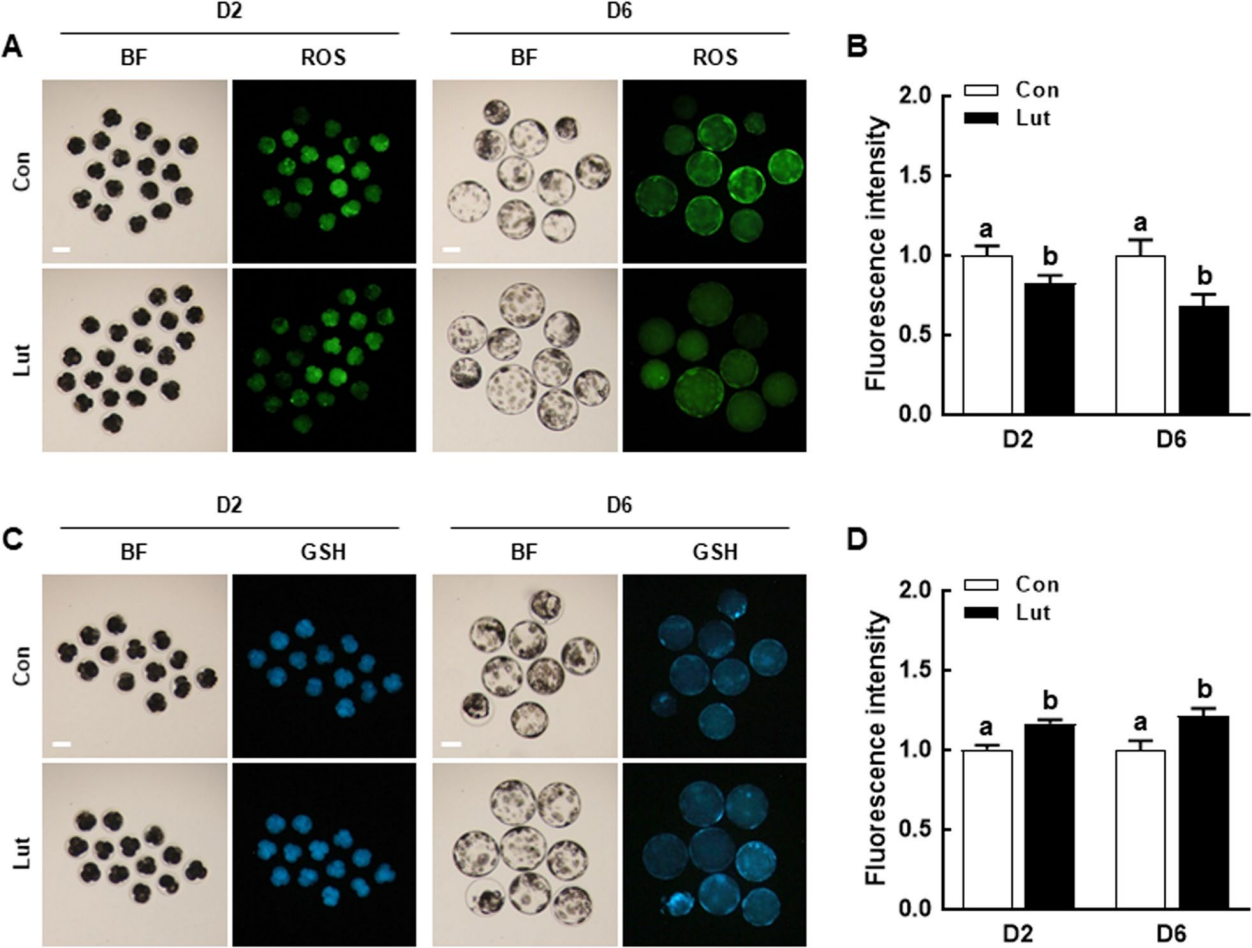
Effects of Lut on intracellular reactive oxygen species (ROS) and glutathione (GSH) levels in porcine PA embryos. **A** Fluorescent images of D2 embryos and D6 blastocysts stained with CM-H2DCFDA in the indicated groups. Scale bar = 100 µm. **B** Quantification of ROS fluorescence intensity in the indicated groups (D2; *n* = 48 per group, D6; *n* = 26 per group). **C** Fluorescent images of D2 embryos and D6 blastocysts stained with CMF2HC in the indicated groups. Scale bar = 100 µm. **D** Quantification of GSH fluorescence intensity in the indicated groups (D2; *n* = 36 per group, D6; *n* = 24 per group). The data are derived from three independent experiments, and means with similar superscripts do not differ (*P* > 0.05)

To evaluate the antioxidant properties of Lut in porcine IVP embryos, we analyzed the intracellular levels of ROS and GSH in D2 embryos and D6 blastocysts. The intracellular ROS levels were significantly lower in D2 embryos and D6 blastocysts that were supplemented with Lut compared to the control (Fig. 2A and B). Conversely, the intracellular GSH levels were significantly higher in D2 embryos and D6 blastocysts with Lut supplementation compared to the control (Fig. 2C and D). These findings indicate that Lut exhibits antioxidant activity during porcine early embryogenesis.

### Lut enhances mitochondrial function in porcine PA embryos

**Fig. 3:**
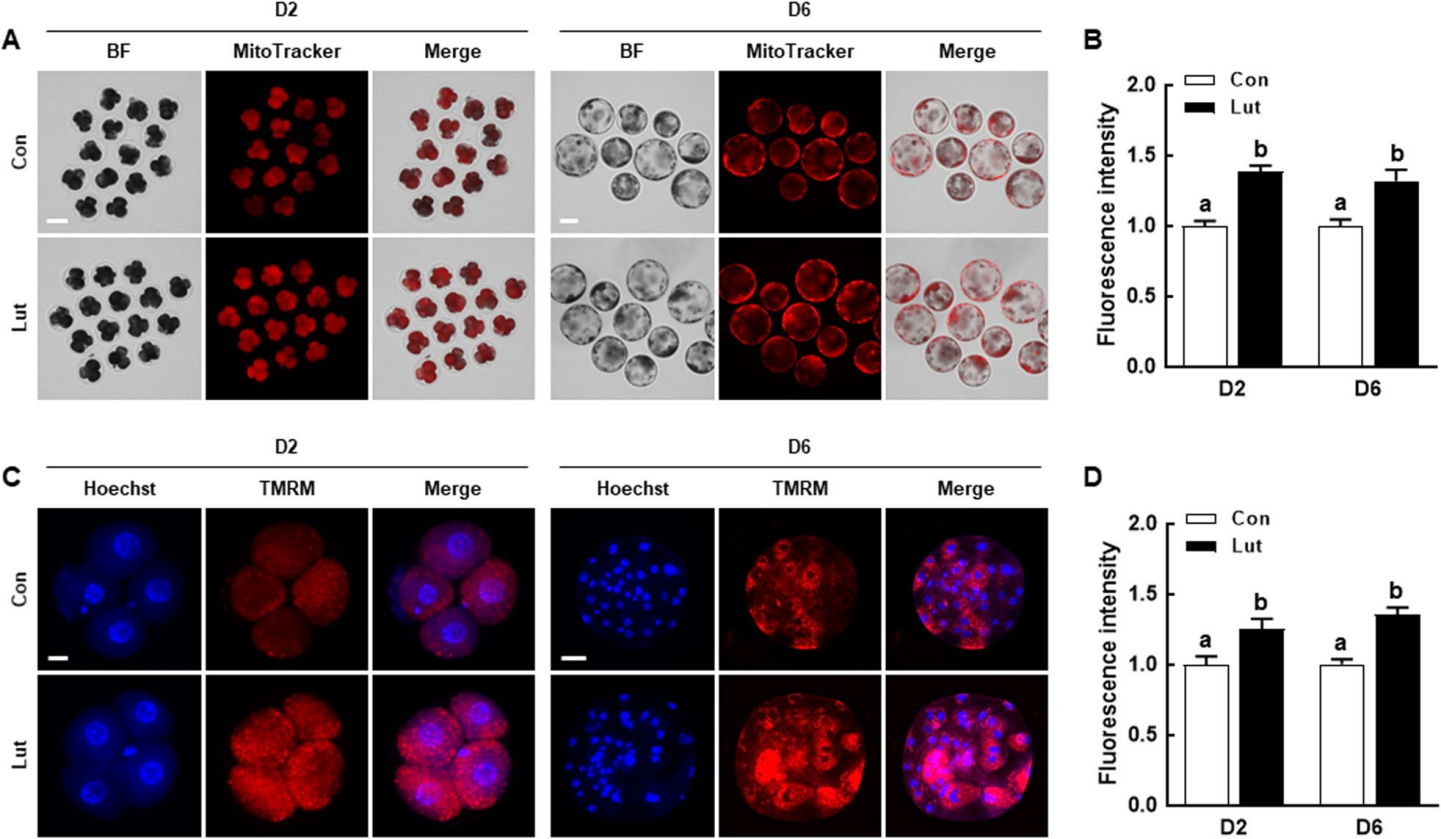
Effects of Lut on mitochondrial functions in porcine PA embryos. **A** Fluorescent images of D2 embryos and D6 blastocysts stained with MitoTracker in the indicated groups. Scale bar = 100 µm. **B** Quantification of MitoTracker fluorescence intensity in the indicated groups (D2; *n* = 51 per group, D6; *n* = 26 per group). **C** Fluorescent images of D2 embryos and D6 blastocysts stained with TMRM in the indicated groups. Scale bar = 50 µm. **D** Quantification of TMRM fluorescence intensity in the indicated groups (D2; *n* = 23 per group, D6; *n* = 24 per group). The data are derived from three independent experiments, and means with similar superscripts do not differ (*P* > 0.05)

To investigate the impact of Lut on mitochondrial function in porcine IVP embryos, we assessed mitochondrial content and membrane potential in D2 embryos and D6 blastocysts using MitoTracker and TMRM staining, respectively. Both the MitoTracker and TMRM intensity levels were significantly elevated in D2 embryos and D6 blastocysts that were supplemented with Lut compared to the control (Fig. 3). These findings suggest that Lut supplementation boosts the developmental competence of porcine IVP embryos by shielding mitochondrial function from ROS-mediated damages.

### Lut activates the Nrf2/Keap1 signaling pathway in porcine PA embryos

**Fig. 4:**
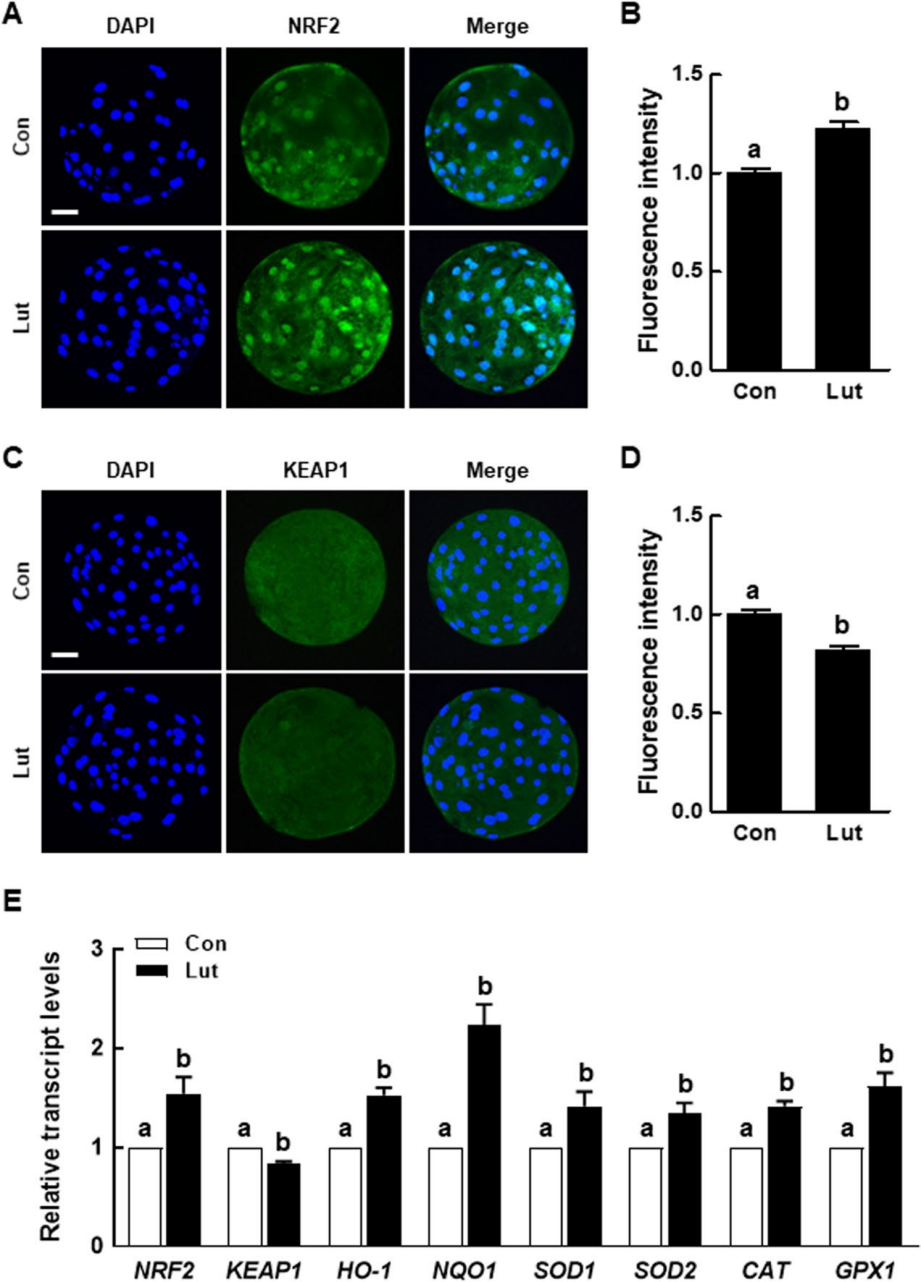
Effects of Lut on the Nrf2/Keap1 pathway in porcine PA embryos. **A** Fluorescent images of blastocysts stained for Nrf2 in the indicated groups. Scale bar = 50 µm. **B** Quantification of Nrf2 fluorescence intensity in the indicated groups (*n* = 24 per group). **C** Fluorescent images of blastocysts stained for Keap1 in the indicated groups. Scale bar = 50 µm. **D** Quantification of fluorescence intensity in the indicated groups (*n* = 30 per group). **E** Quantitative RT-PCR results for Nrf2/Keap1 signaling pathway-related genes in blastocysts (*n* = 3 per group). The data are derived from three independent experiments, and means with similar superscripts do not differ (*P* > 0.05)

The Nrf2/Keap1 signaling pathway is renowned for its role in cellular defense mechanisms against oxidative stress. To determine whether Lut activates the Nrf2/Keap1 signaling pathway during porcine early embryogenesis, we examined the expression levels of proteins and mRNAs associated with the Nrf2/Keap1 signaling pathway in porcine blastocysts. The Nrf2 levels were significantly higher, while the Keap1 levels were notably lower in the Lut group compared to the control (Fig. 4A–D). Furthermore, Lut supplementation altered the expression levels of downstream target genes associated with the Nrf2/Keap1 signaling pathway (Fig. 4E), suggesting that Lut activates this pathway during porcine early embryogenesis. Additionally, we cultured PA embryos in a culture medium treated with various concentrations of brusatol (an Nrf2 inhibitor; 0, 20, 50, and 100 nmol/L) for 6 d. While brusatol did not influence the cleavage rate, it significantly diminished the blastocyst formation rate and total cell number in a concentration-dependent manner (Additional file 6: Table S6, Additional file 10: Fig. S2). Consequently, we selected 50 nmol/L brusatol for subsequent experiments.

### Lut counteracts the detrimental effects of brusatol on the developmental competence of porcine PA embryos

**Fig. 5:**
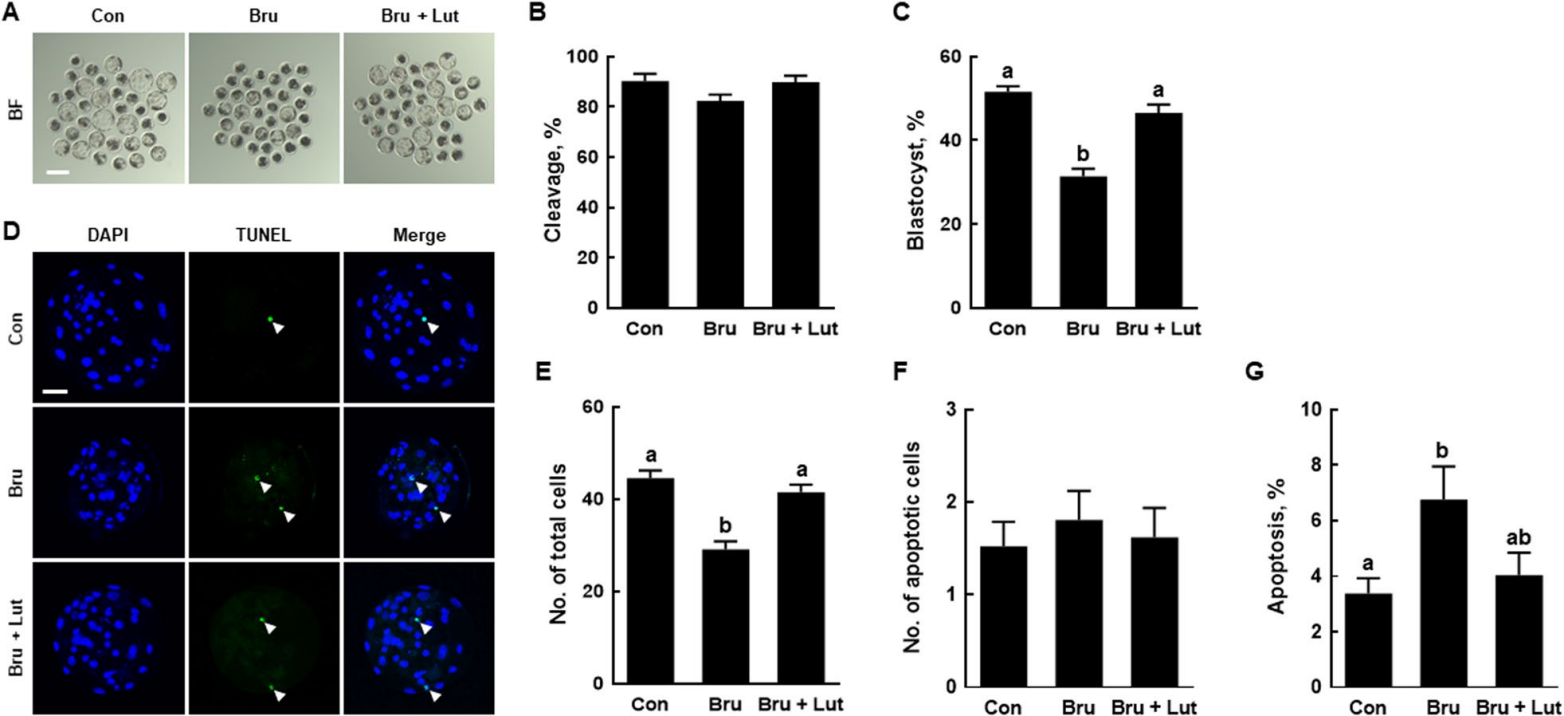
Role of the Nrf2/Keap1 pathway in the effects of Lut on the early development of porcine PA embryos. **A** Representative bright-field images of blastocysts cultured in the indicated groups. Scale bar = 200 µm. **B**, **C** Quantification of the cleavage rate and blastocyst formation rate in the indicated groups (Con; *n* = 180, Bru; *n* = 180, Bru + Lut; *n* = 181). **D** TUNEL assay of blastocysts in the indicated groups. Embryos were stained for TUNEL (green, white arrow) and nuclei (blue). Scale bar = 50 µm. **E**-**G** Quantification of the total cell number, number of apoptotic cells, and proportion of apoptotic cells in the indicated groups (*n* = 21 per group). The data are derived from five independent experiments, and means with similar superscripts do not differ (*P* > 0.05)

**Fig. 6:**
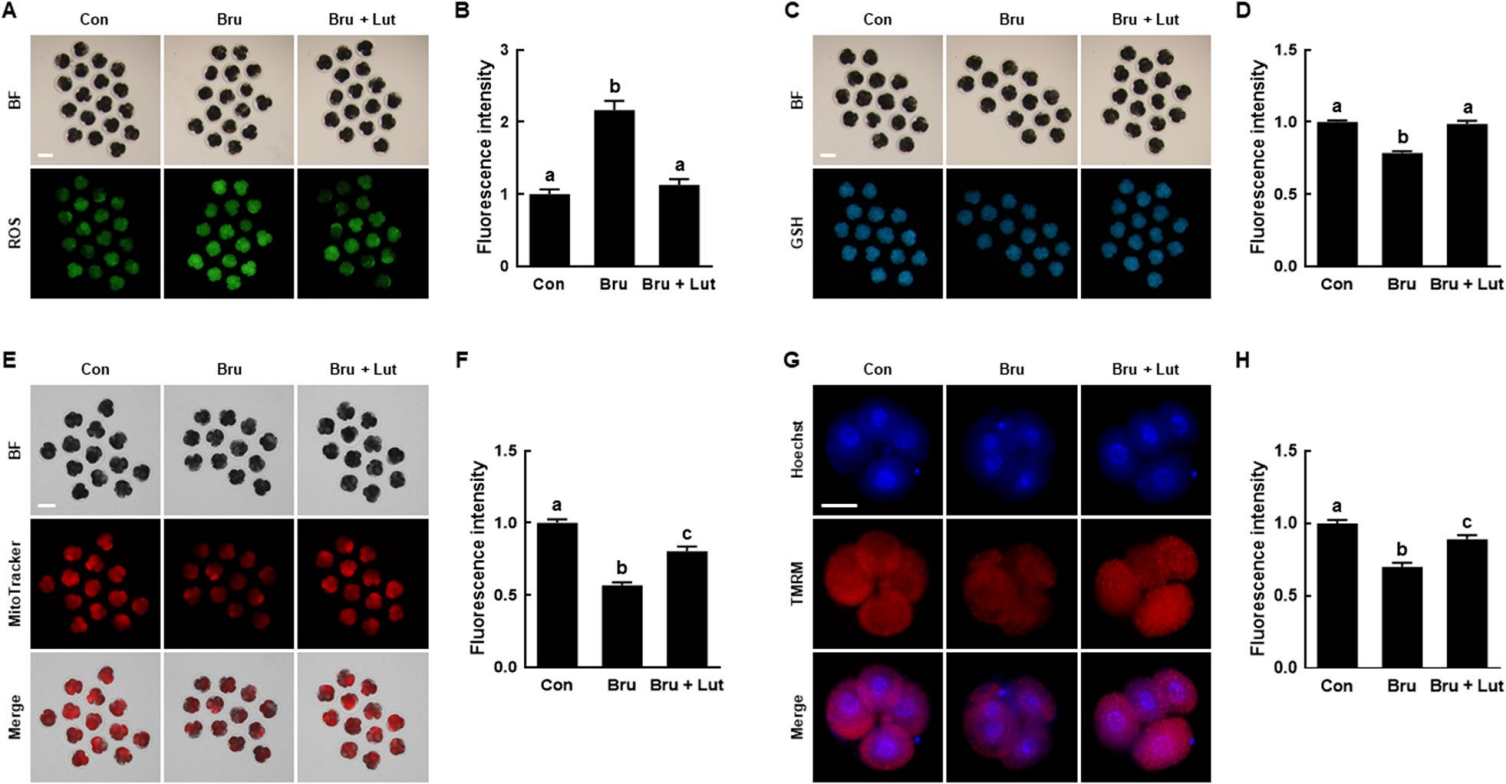
Role of the Nrf2/Keap1 pathway in the effects of Lut on ROS, GSH, and mitochondrial function in porcine PA embryos. **A** Fluorescent images of embryos stained with CM-H2DCFDA in the indicated groups. Scale bar = 100 µm. **B** Quantification of ROS fluorescence intensity in the indicated groups (*n* = 36 per group). **C** Fluorescent images of embryos stained with CMF2HC in the indicated groups. Scale bar = 100 µm. **D** Quantification of GSH fluorescence intensity in the indicated groups (*n* = 38 per group). **E** Fluorescent images of embryos stained with MitoTracker in the indicated groups. Scale bar = 100 µm. **F** Quantification of MitoTracker fluorescence intensity in the indicated groups (*n* = 42 per group). **G** Fluorescent images of embryos stained with TMRM in the indicated groups. Scale bar = 50 µm. **H** Quantification of TMRM fluorescence intensity in the indicated groups (*n* = 24 per group). The data are derived from three independent experiments, and means with similar superscripts do not differ (*P* > 0.05)

To elucidate the interplay between the Nrf2/Keap1 signaling pathway and the beneficial effects of Lut, we cultured PA embryos in IVC medium supplemented with brusatol, with or without Lut, and evaluated the cleavage rate, blastocyst formation rate, and apoptosis rate. The reduction in the blastocyst formation rate caused by brusatol exposure was alleviated by Lut supplementation, restoring it to control levels, although it did not influence the cleavage rate (Fig. 5A–C, Additional file 7: Table S7). Additionally, from the TUNEL assay, we observed that the Lut supplementation ameliorated the brusatol-induced decrease in total cell number and increase in apoptosis rate (Fig. 5D–G, Additional file 8: Table S8). To further substantiate these observations, we assessed intracellular ROS and GSH levels. Intracellular ROS levels were significantly elevated, and GSH levels were markedly diminished in D2 embryos supplemented with brusatol. However, Lut supplementation reestablished this imbalance to control levels (Fig. 6A–D). Moreover, the decrease in mitochondrial content and membrane potential induced by brusatol exposure was also counteracted by Lut supplementation (Fig. 6E–H).

## Discussion

**Fig. 7:**
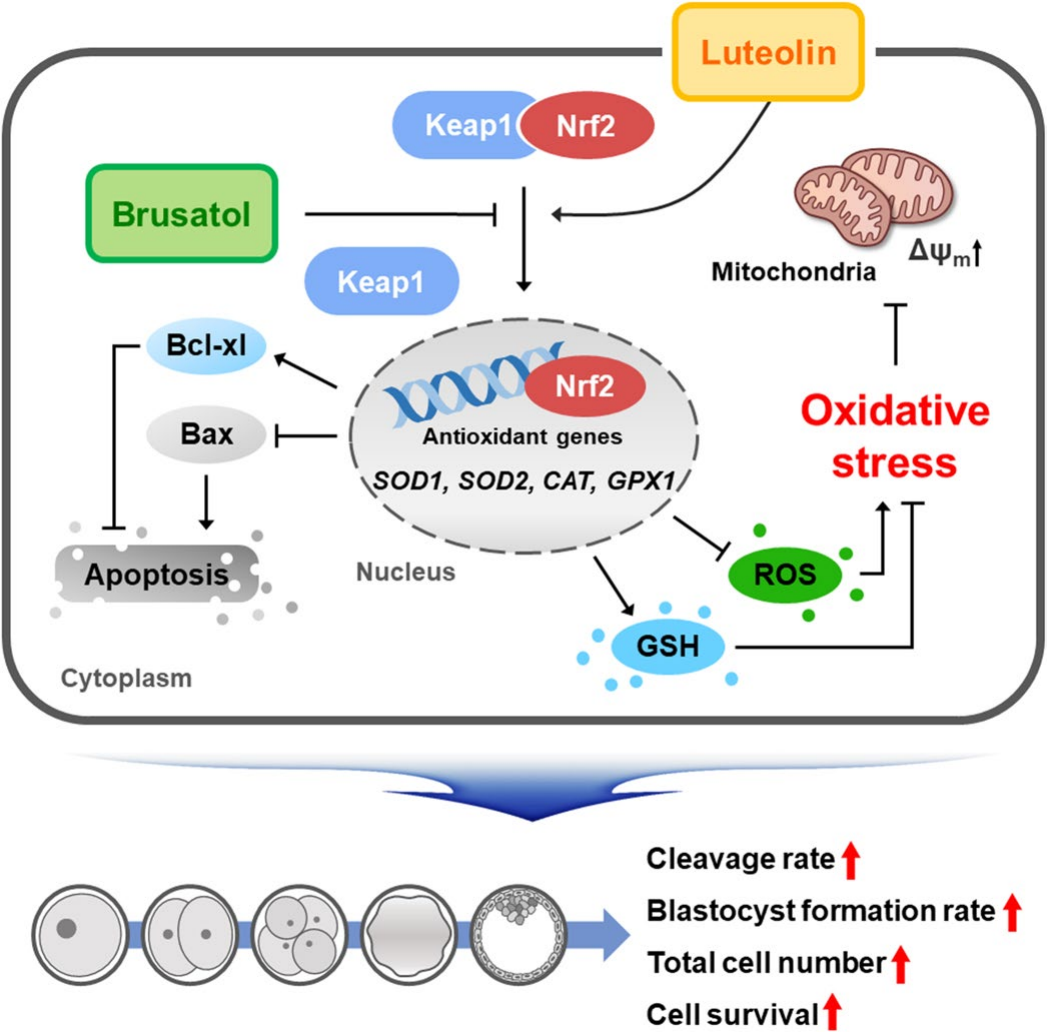
Graphical overview of the beneficial effects of Lut on porcine early embryogenesis. Lut supplementation during in vitro culture increases cleavage rate, blastocyst formation rate, total cell number, and cellular survival in porcine in vitro produced (IVP) embryos. Additionally, Lut exhibits antioxidant activity and enhances mitochondrial function. Notably, Lut activates the Nrf2/Keap1 signaling pathway, and the impairments in early embryogenesis caused by brusatol are alleviated by Lut supplementation. These results suggest that Lut exerts beneficial effects on the developmental competence of porcine IVP embryos by reducing oxidative stress through the activation of the Nrf2/Keap1 signaling pathway

During IVC, oxidative stress can hinder the developmental competence of porcine IVP embryos, affecting parameters such as blastocyst formation, cell numbers, and cellular survival. Although considerable efforts have been made to enhance IVC systems by employing various antioxidants, the quality of porcine IVP embryos still falls short in comparison to in vivo-derived embryos [12, 24]. This underscores the need for continued exploration of more effective antioxidants and the optimization of IVC systems to both improve the quality of porcine IVP embryos and uncover the mechanisms involved. In this study, we delved into the effects of Lut on porcine IVP embryos and probed the potential underlying mechanisms. Our findings reveal that Lut ameliorates the developmental competence of porcine IVP embryos against oxidative stress, which is mediated through the activation of the Nrf2/Keap1 signaling pathway (Fig. 7).

Lut, a yellow dye compound extracted from the plant *Reseda luteola*, contributes to the plant's ability to counter oxidative stress. Its antioxidant activity has been attributed to several mechanisms [14]. Structurally, Lut scavenges ROS through its oxidation, stabilizing the radical group, an ability attributed to the presence of a double bond between C2 and C3, a carbonyl group on C4, and 3′,4′ hydroxylation [25]. Furthermore, Lut curtails ROS generation by chelating transition metal ions and restraining xanthine oxidase activity [26]. It also directly inhibits enzymes responsible for the oxidation of cellular components, including lipoxygenase and cyclooxygenase [27]. Additionally, Lut can augment antioxidant activity synergistically through interactions with other antioxidants, such as vitamins and the cellular redox system [28]. Prior studies have highlighted effects of Lut in attenuating oxidative damage and mitigating free radical formation in rat cortical cells and neuroblastoma cells [29, 30]. Notably, our previous studies have shown that Lut can improve porcine oocyte quality and subsequent embryonic development by alleviating oxidative damage across various organelle dynamics [31, 32]. In the present study, we observed that embryos supplemented with Lut exhibited significantly lower levels of ROS and higher levels of GSH compared to the control. Furthermore, the addition of Lut during IVC markedly enhanced the developmental competence, as evidenced by the increase in cleavage rate, blastocyst formation rate, cell numbers, and cellular survival in porcine PA and IVF embryos. These findings underscore the potential of Lut as an antioxidant agent in porcine IVC systems.

Building on the understanding of Lut's antioxidant activity, we turned our focus to its relationship with the Nrf2/Keap1 signaling pathway, which is instrumental in maintaining cellular redox homeostasis through the activation of antioxidant enzymes. An accumulating body of evidence underscores the significance of the Nrf2/Keap1 signaling pathway within the female reproductive system. For instance, Nrf2 knockout mice display accelerated ovarian failure, a shortened gestation period, diminished birth weight, and increased cellular apoptosis [33, 34]. Moreover, silencing Nrf2 through the injection of small interfering RNA into fully grown oocytes adversely affects the maintenance of spindle assembly and meiotic division [35]. While previous studies have documented that Lut counteracts oxidative stress by fortifying the Nrf2/Keap1 signaling pathway in diverse cell types [21, 36], no studies have yet explored the impact of Lut on this signaling pathway in the context of porcine early embryogenesis. Our findings reveal that the inclusion of Lut in the IVC significantly elevates Nrf2 levels and the abundance of downstream antioxidant target mRNAs, while concurrently decreasing Keap1 levels. This suggests that Lut may thwart proteasomal degradation of the Nrf2 protein by interfering with the Keap1-dependent E3 ubiquitin activity, culminating in the stabilization and nuclear accumulation of Nrf2. Additionally, Nrf2 is identified as an essential regulator of self-renewal and pluripotency in human embryonic stem cells [37]. Previous studies have posited that the inclusion of antioxidants in IVC bolsters blastocyst formation rate, cell numbers, and pluripotency by mitigating oxidative stress via the activation of the Nrf2/Keap1 signaling pathway in porcine IVP embryos [38, 39]. In alignment with these studies, our data reveals that Lut supplementation during IVC significantly elevates both the developmental competence and the expression levels of developmental potential-related genes, which suggests that Nrf2 is instrumental in governing pluripotency during cellular reprogramming. These findings compellingly indicate that the Nrf2/Keap1 signaling pathway is central to porcine early embryogenesis.

To further ascertain whether Lut's antioxidant activity enhances the developmental competence of porcine IVP embryos by modulating the Nrf2/Keap1 signaling pathway, we employed brusatol, a specific inhibitor of Nrf2. Brusatol induces a rapid and transient depletion of Nrf2 proteins via post-transcriptional mechanisms, such as the inhibition of protein synthesis and the stimulation of ubiquitination and proteolysis [40]. Previous studies have shown that treatment with brusatol impairs mouse embryonic development by inhibiting cell cycle progression; however, these adverse effects can be ameliorated by microinjection of an Nrf2 overexpression plasmid [41]. Furthermore, brusatol treatment has been found to substantially decrease blastocyst formation, total cell number, and cellular survival in porcine embryos [42]. Consistent with these studies, our data demonstrates that brusatol substantially and concentration-dependently reduces the blastocyst formation rate and total cell number. Intriguingly, Lut supplementation was able to mitigate the detrimental effects of brusatol on porcine early embryogenesis, underscoring the intimate link between beneficial effects of Lut and the Nrf2/Keap1 signaling pathway.

Mitochondria are integral organelles for the successful maturation of oocytes, fertilization, and embryonic development, owing to their role in regulating cellular energy metabolism, calcium signaling, and apoptosis [43]. Consequently, mitochondrial membrane potential is extensively employed as an indicator of mitochondrial function. Nonetheless, excessive accumulation of ROS can inhibit the process of mitochondrial biogenesis, which encompasses the synthesis of new mtDNA, membrane formation, and mitochondrial division [44]. A study has revealed that Lut fosters mitochondrial biogenesis by amplifying the levels of peroxisome proliferator-activated receptor γ coactivator 1α, a principal orchestrator of mitochondrial biogenesis, and nitric oxide levels, which sets in motion signaling events that underpin mitochondrial biogenesis [45]. Additionally, Lut stimulates sirtuins and forkhead box O3a, which are situated in the mitochondria and modulate metabolism by curbing ROS production [46]. In alignment with previous studies, our findings indicate that Lut supplementation during IVC augments mitochondrial content and membrane potential in porcine IVP embryos, signifying that Lut bolsters mitochondrial function by alleviating oxidative stress. It is noteworthy that one of the salient functions of Nrf2 is to oversee mitochondrial biogenesis and functionality by enhancing the antioxidant response [47]. Nrf2 boosts mitochondrial membrane potential for ATP production by promoting glucose uptake and pyruvate flux [48]. Our findings demonstrated that Lut supplementation counteracts the depletion of mitochondrial content and membrane potential induced by brusatol exposure. Collectively, these findings suggest that Lut exerts protective effects on mitochondrial biogenesis and ameliorates mitochondrial dysfunction by activating the Nrf2/Keap1 signaling pathway.

## Conclusion

This study represents the first investigation into the potential association between Lut and the Nrf2/Keap1 signaling pathway in porcine IVP embryos. These results may offer a promising strategy for enhancing porcine IVC systems and afford insights into the role of the Nrf2/Keap1 signaling pathway in porcine early embryogenesis. Our findings can be applied to obtain a higher yield from assisted reproductive technologies as a method to improve pig production and also as a basic tool for preserving endangered porcine breeds.

### Supplementary Information

**Additional file 1:Table S1.** Primer sequences for qRT-PCR.

**Additional file 2:Table S2.** Effects of Luteolin (Lut) concentrations on in vitro development of porcine parthenogenetic activation (PA) embryos.

**Additional file 3:Table S3.** Effects of Lut on cell survival in porcine PA blastocysts.

**Additional file 4:Table S4.** Effects of Luteolin (Lut) concentrations on in vitro development of porcine *in vitro* fertilization (IVF) embryos.

**Additional file 5:Table S5.** Effects of Lut on cell survival in porcine IVF blastocysts.

**Additional file 6:Table S6.** Effects of Brusatol (Bru) concentrations on in vitro development of porcine PA embryos.

**Additional file 7:Table S7.** Co-treatment effects of Bru and Lut on in vitro development of porcine PA embryos.

**Additional file 8:Table S8.** Co-treatment effects of Bru and Lut on cell survival in porcine PA blastocysts.

**Additional file 9:Fig. S1.** Effects of Lut on the developmental competence of porcine in vitro fertilization embryos. **A** Representative bright-field images (upper, scale bar = 200 µm) and nuclear-stained images (lower, scale bar = 50 µm) of blastocysts cultured in the presence or absence of Lut. **B**–**D** Quantification of cleavage rate, blastocyst formation rate, and total cell number in the indicated groups (0; *n* = 214, 0.5; *n* = 216). **E** TUNEL assay of blastocysts in the indicated groups. Embryos were stained for TUNEL (green, indicated by white arrows) and nuclei (blue). Scale bar = 50 µm. **F**, **G** Quantification of the number and proportion of apoptotic cells in the indicated groups (*n* = 34 per group). The data are derived from three independent experiments, and means with similar superscripts do not differ (*P* > 0.05).

**Additional file 10:Fig. S2. **Effects of Brusatol on Developmental Competence of Porcine PA Embryos. **A** Representative bright-field (upper, scale bar = 200 µm) and nuclear-stained (lower, scale bar = 50 µm) images of blastocysts cultured with or without varying concentrations of brusatol. **B**-**D** Quantification of the cleavage rate, blastocyst formation rate, and total cell number in the indicated groups (0; *n* = 181, 20; *n* = 182, 50; *n* = 181, 100; *n* = 181). The data are derived from five independent experiments, and means with similar superscripts do not differ (*P* > 0.05).

## Data Availability

The data that support the findings of this study are available from the corresponding author upon reasonable request.

## Abbreviations

ARE: Antioxidant response element
BSA: Bovine serum albumin
CAT: Catalase
COCs: Cumulus-oocyte complexes
DMSO: Dimethyl sulfoxide
GPX: Glutathione peroxidase
GSH: Glutathione
IVC: In vitro culture
IVF: In vitro fertilization
IVM: In vitro maturation
IVP: In vitro production
Keap1: Kelch-like ECH-associated protein 1
Lut: Luteolin
mTBM: Modified Tris-buffered medium
Nrf2: Erythroid-2-related factor 2
PA: Parthenogenetic activation
PVA-PBS: 0.1% Polyvinyl alcohol
ROS: Reactive oxygen species
RT: Room temperature
SOD: Superoxide dismutase
TUNEL: Terminal Deoxynucleotidyl Transferase-Mediated dUTP-Digoxygenin Nick End-Labeling
