# Supplementary material for: Enhancement of porcine in vitro embryonic development through luteolin-mediated activation of the Nrf2/Keap1 signaling pathway
Source: J Anim Sci Biotechnol. 2023 Dec 1;14:148. doi: 10.1186/s40104-023-00947-9 (PMC10691000; doi:10.1186/s40104-023-00947-9)
Supplement: Supplementary file 1 — Additional file 1:Table S1. Primer sequences for qRT-PCR. [file 40104_2023_947_MOESM1_ESM.doc]

**Table S1** Primer sequences for qRT-PCR

| **Gene** | **Primer sequences** | **GenBank**  **accession no.** | **Product**  **Size, bp** |
| --- | --- | --- | --- |
| *OCT4* | F: 5’- AGTGAGAGGCAACCTGGAGA -3’ | NM_001113060.1 | 151 |
|  | R: 5’- ACTGCTTGATCGTTTGCCCT -3’ |  |  |
| *CDX2* | F: 5’- GGCAGCCAAGTGAAAACCAG -3’ | NM_001278769.1 | 119 |
|  | R: 5’- GCCTTTCTCCGAATGGTGAT -3’ |  |  |
| *BAX* | F: 5’- CGATCTCGAAGGAAGTCCAG -3’ | XM_003127290.5 | 251 |
|  | R: 5’- AAGCGCATTGGAGATGAACT -3’ |  |  |
| *BCL-XL* | F: 5’- AGGGCATTCAGTGACCTGAC -3’ | NM_214285.1 | 242 |
|  | R: 5’- TGGATCCAAGGCTCTAGG TG -3’ |  |  |
| *NRF2* | F: 5’- CCTTCTGGGGATACAGTCCA -3’ | XM_005671981.3 | 110 |
|  | R: 5’- CCGGGACTTATAGGCACTTC -3’ |  |  |
| *KEAP1* | F: 5’- GCCTCATCGAGTTCGCTTAC -3’ | NM_001114671.1 | 105 |
|  | R: 5’- CACGGACCACACTGTCAATC -3’ |  |  |
| *HO-1* | F: 5’- CCTTTTGACGTGCCTTGAT -3’ | NM_001004027.1 | 114 |
|  | R: 5’- GAACGAAGAGTGGCTCCAAC -3’ |  |  |
| *NQO1* | F: 5’- GTCTTTCTGTGGGCCATCAC -3’ | NM_001159613.1 | 146 |
|  | R: 5’- CGAAAGCAAGTCAAGGAAGG -3’ |  |  |
| *SOD1* | F: 5’- GGTGGGCCAAAGGATCAAGA -3’ | NM_001190422.1 | 80 |
|  | R: 5’- TACACAGTGGCCACACCATC -3’ |  |  |
| *SOD2* | F: 5’- GGTGGAGGCCACATCAATCA -3’ | NM_214127.2 | 220 |
|  | R: 5’- AACAAGCGGCAATCTGCAAG -3’ |  |  |
| *CAT* | F: 5’- TGTACCCGCTATTCTGGGGA -3’ | NM_214301.2 | 119 |
|  | R: 5’- TCACACAGGCGTTTCCTCTC -3’ |  |  |
| *GPX1* | F: 5’- TGGACATCAGGAAAATGCCAAG -3’ | NM_214201.1 | 127 |
|  | R: 5’- GTGAGCATTTGCGCCATTCA -3’ |  |  |
| *H2A* | F: 5’- AGTTTCCTGTGGGTCGAGTG -3’ | NM_001244473 | 162 |
|  | R: 5’- TGCGAGTCTTCTTGTTGTC -3’ |  |  |
