# Supplementary material for: Enhancement of porcine in vitro embryonic development through luteolin-mediated activation of the Nrf2/Keap1 signaling pathway
Source: J Anim Sci Biotechnol. 2023 Dec 1;14:148. doi: 10.1186/s40104-023-00947-9 (PMC10691000; doi:10.1186/s40104-023-00947-9)
Supplement: Supplementary file 2 — Additional file 2:Table S2. Effects of Luteolin (Lut) concentrations on in vitro development of porcine parthenogenetic activation (PA) embryos. [file 40104_2023_947_MOESM2_ESM.doc]

**Table S2** Effects of Luteolin (Lut) concentrations on in vitro development of porcine parthenogenetic activation (PA) embryos

| **Luteolin, μmol/L** | **No. of embryos examined** | **Cleavage, %** | **Blastocyst, %** | **Total cell number** |
| --- | --- | --- | --- | --- |
| 0 | 197 | 161 (81.7 ± 2.0)^a^ | 113 (57.5 ± 3.7)^a^ | 33.5 ± 1.8^a^ |
| 0.05 | 196 | 175 (89.1 ± 2.5)^ab^ | 129 (65.4 ± 4.8)^ab^ | 36.7 ± 1.8^ab^ |
| 0.5 | 195 | 175 (89.9 ± 2.0)^b^ | 142 (72.8 ± 1.4)^b^ | 40.7 ± 1.3^b^ |
| 5 | 195 | 162 (83.0 ± 1.3)^ab^ | 115 (58.9 ± 1.5)^a^ | 32.6 ± 1.1^a^ |

Data are the mean ± SEM, and values with different superscript letter within a column differ significantly (*P* < 0.05)
