# Supplementary material for: Enhancement of porcine in vitro embryonic development through luteolin-mediated activation of the Nrf2/Keap1 signaling pathway
Source: J Anim Sci Biotechnol. 2023 Dec 1;14:148. doi: 10.1186/s40104-023-00947-9 (PMC10691000; doi:10.1186/s40104-023-00947-9)
Supplement: Supplementary file 3 — Additional file 3:Table S3. Effects of Lut on cell survival in porcine PA blastocysts. [file 40104_2023_947_MOESM3_ESM.doc]

**Table S3** Effects of Lut on cell survival in porcine PA blastocysts

| **Groups** | **No. of blastocysts examined** | **No. of TUNEL-positive cells** | **Apoptosis, %** |
| --- | --- | --- | --- |
| Con | 42 | 2.4 ± 0.2^a^ | 6.7 ± 0.9^a^ |
| Lut | 42 | 1.8 ± 0.2^b^ | 4.6 ± 0.4^b^ |

Data are the mean ± SEM, and values with different superscript letter within a column differ significantly (*P* < 0.05)
