# Supplementary material for: Enhancement of porcine in vitro embryonic development through luteolin-mediated activation of the Nrf2/Keap1 signaling pathway
Source: J Anim Sci Biotechnol. 2023 Dec 1;14:148. doi: 10.1186/s40104-023-00947-9 (PMC10691000; doi:10.1186/s40104-023-00947-9)
Supplement: Supplementary file 4 — Additional file 4:Table S4. Effects of Luteolin (Lut) concentrations on in vitro development of porcine in vitro fertilization (IVF) embryos. [file 40104_2023_947_MOESM4_ESM.doc]

**Table S4** Effects of Luteolin (Lut) concentrations on in vitro development of porcine *in vitro* fertilization (IVF) embryos

| **Groups** | **No. of embryos examined** | **Cleavage, %** | **Blastocyst, %** | **Total cell number** |
| --- | --- | --- | --- | --- |
| Con | 214 | 176 (82.5 ± 1.4)^a^ | 97 (44.9 ± 2.9)^a^ | 40.5 ± 1.6^a^ |
| Lut | 216 | 195 (89.7 ± 2.5)^b^ | 124 (56.8 ± 3.9)^b^ | 46.0 ± 1.7^b^ |

Data are the mean ± SEM, and values with different superscript letter within a column differ significantly (*P* < 0.05)
