# Supplementary material for: Enhancement of porcine in vitro embryonic development through luteolin-mediated activation of the Nrf2/Keap1 signaling pathway
Source: J Anim Sci Biotechnol. 2023 Dec 1;14:148. doi: 10.1186/s40104-023-00947-9 (PMC10691000; doi:10.1186/s40104-023-00947-9)
Supplement: Supplementary file 5 — Additional file 5:Table S5. Effects of Lut on cell survival in porcine IVF blastocysts. [file 40104_2023_947_MOESM5_ESM.doc]

**Table S5** Effects of Lut on cell survival in porcine IVF blastocysts

| **Groups** | **No. of blastocysts examined** | **No. of TUNEL-positive cells** | **Apoptosis, %** |
| --- | --- | --- | --- |
| Con | 34 | 3.5 ± 0.3^a^ | 8.8 ± 0.7^a^ |
| Lut | 34 | 2.6 ± 0.3^b^ | 5.6 ± 0.6^b^ |

Data are the mean ± SEM, and values with different superscript letter within a column differ significantly (*P* < 0.05)
