# Supplementary material for: Enhancement of porcine in vitro embryonic development through luteolin-mediated activation of the Nrf2/Keap1 signaling pathway
Source: J Anim Sci Biotechnol. 2023 Dec 1;14:148. doi: 10.1186/s40104-023-00947-9 (PMC10691000; doi:10.1186/s40104-023-00947-9)
Supplement: Supplementary file 6 — Additional file 6:Table S6. Effects of Brusatol (Bru) concentrations on in vitro development of porcine PA embryos. [file 40104_2023_947_MOESM6_ESM.doc]

**Table S6** Effects of Brusatol (Bru) concentrations on in vitro development of porcine PA embryos

| **Brusatol, nmol/L** | **No. of embryos examined** | **Cleavage, %** | **Blastocyst, %** | **Total cell number** |
| --- | --- | --- | --- | --- |
| 0 | 181 | 161 (89.0 ± 2.5) | 113 (62.5 ± 2.3)^a^ | 44.1 ± 1.9^a^ |
| 20 | 182 | 163 (89.3 ± 2.7) | 111 (60.7 ± 2.6)^a^ | 35.1 ± 1.7^b^ |
| 50 | 181 | 161 (88.7 ± 2.9) | 77 (42.5 ± 3.8)^b^ | 31.0 ± 1.3^b^ |
| 100 | 181 | 161 (89.0 ± 3.0) | 35 (19.2 ± 3.8)^c^ | 25.0 ± 0.8^c^ |

Data are the mean ± SEM, and values with different superscript letter within a column differ significantly (*P* < 0.05)
