# Supplementary material for: Enhancement of porcine in vitro embryonic development through luteolin-mediated activation of the Nrf2/Keap1 signaling pathway
Source: J Anim Sci Biotechnol. 2023 Dec 1;14:148. doi: 10.1186/s40104-023-00947-9 (PMC10691000; doi:10.1186/s40104-023-00947-9)
Supplement: Supplementary file 7 — Additional file 7:Table S7. Co-treatment effects of Bru and Lut on in vitro development of porcine PA embryos. [file 40104_2023_947_MOESM7_ESM.doc]

**Table S7** Co-treatment effects of Bru and Lut on in vitro development of porcine PA embryos

| **Groups** | **No. of embryos examined** | **Cleavage, %** | **Blastocyst, %** | **Total cell number** |
| --- | --- | --- | --- | --- |
| Con | 180 | 161 (90.3 ± 2.8) | 93 (51.6 ± 1.3)^a^ | 44.6 ± 1.6^a^ |
| Bru | 180 | 163 (82.5 ± 2.4) | 56 (31.3 ± 1.9)^b^ | 29.1 ± 1.7^b^ |
| Bru + Lut | 181 | 161 (89.9 ± 2.6) | 84 (46.6 ± 2.0)^a^ | 41.5 ± 1.6^a^ |

Data are the mean ± SEM, and values with different superscript letter within a column differ significantly (*P* < 0.05)
