# Supplementary material for: Enhancement of porcine in vitro embryonic development through luteolin-mediated activation of the Nrf2/Keap1 signaling pathway
Source: J Anim Sci Biotechnol. 2023 Dec 1;14:148. doi: 10.1186/s40104-023-00947-9 (PMC10691000; doi:10.1186/s40104-023-00947-9)
Supplement: Supplementary file 8 — Additional file 8:Table S8. Co-treatment effects of Bru and Lut on cell survival in porcine PA blastocysts. [file 40104_2023_947_MOESM8_ESM.doc]

**Table S8** Co-treatment effects of Bru and Lut on cell survival in porcine PA blastocysts

| **Groups** | **No. of blastocysts examined** | **No. of TUNEL-positive cells** | **Apoptosis, %** |
| --- | --- | --- | --- |
| Con | 21 | 1.5 ± 0.3 | 3.4 ± 0.5^a^ |
| Bru | 21 | 1.8 ± 0.3 | 6.8 ± 1.2 ^b^ |
| Bru + Lut | 21 | 1.6 ± 0.3 | 4.0 ± 0.8^ab^ |

Data are the mean ± SEM, and values with different superscript letter within a column differ significantly (*P* < 0.05)
