# Supplementary figures and images for: Enhancement of porcine in vitro embryonic development through luteolin-mediated activation of the Nrf2/Keap1 signaling pathway
Source: J Anim Sci Biotechnol. 2023 Dec 1;14:148. doi: 10.1186/s40104-023-00947-9 (PMC10691000; doi:10.1186/s40104-023-00947-9)

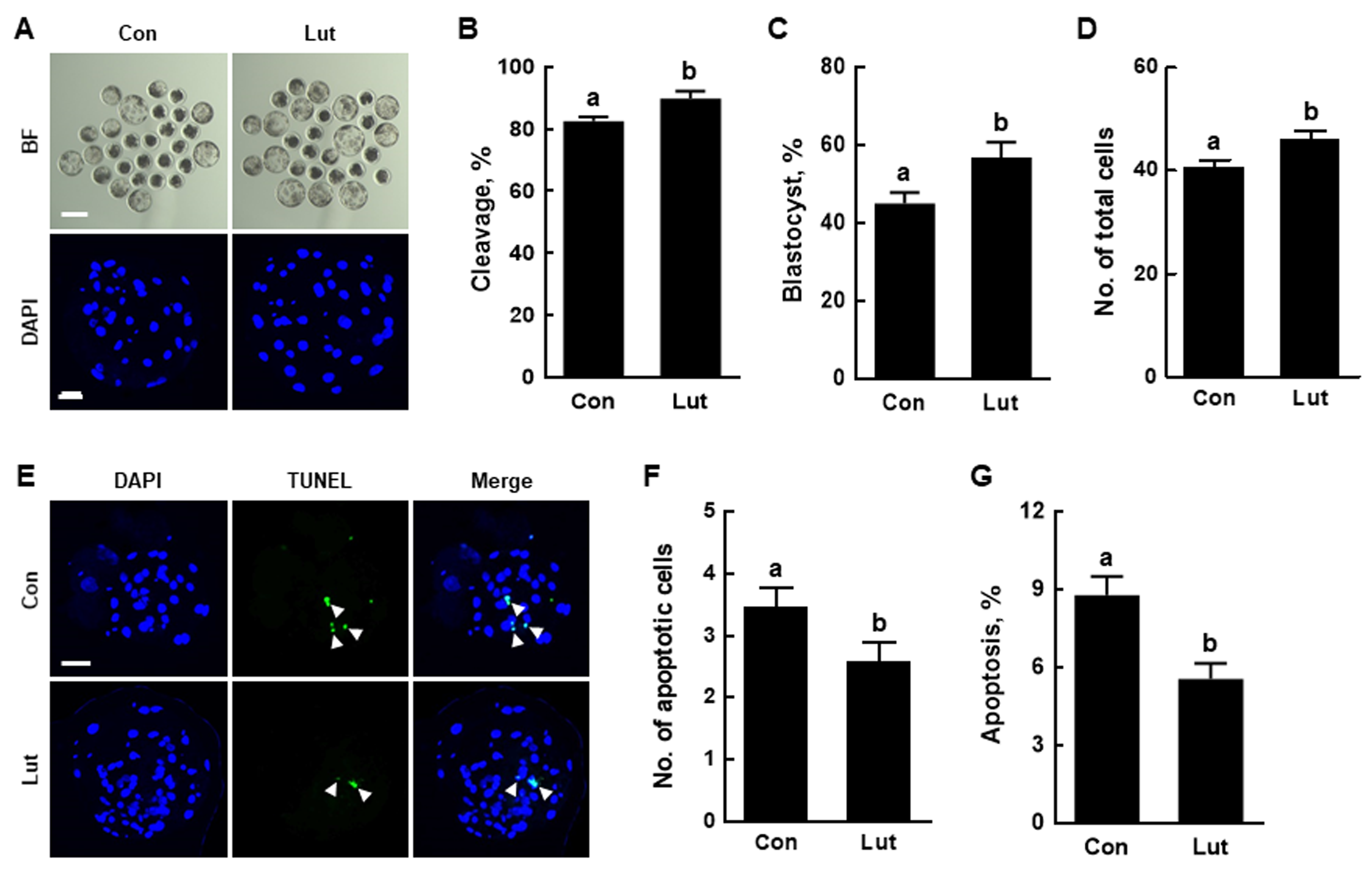

Supplement: Supplementary file 9 — Additional file 9:Fig. S1. Effects of Lut on the developmental competence of porcine in vitro fertilization embryos. A Representative bright-field images (upper, scale bar = 200 µm) and nuclear-stained images (lower, scale bar = 50 µm) of blastocysts cultured in the presence or absence of Lut. B–D Quantification of cleavage rate, blastocyst formation rate, and total cell number in the indicated groups (0; n = 214, 0.5; n = 216). E TUNEL assay of blastocysts in the indicated groups. Embryos were stained for TUNEL (green, indicated by white arrows) and nuclei (blue). Scale bar = 50 µm. F, G Quantification of the number and proportion of apoptotic cells in the indicated groups (n = 34 per group). The data are derived from three independent experiments, and means with similar superscripts do not differ (P > 0.05). [file 40104_2023_947_MOESM9_ESM.tif]

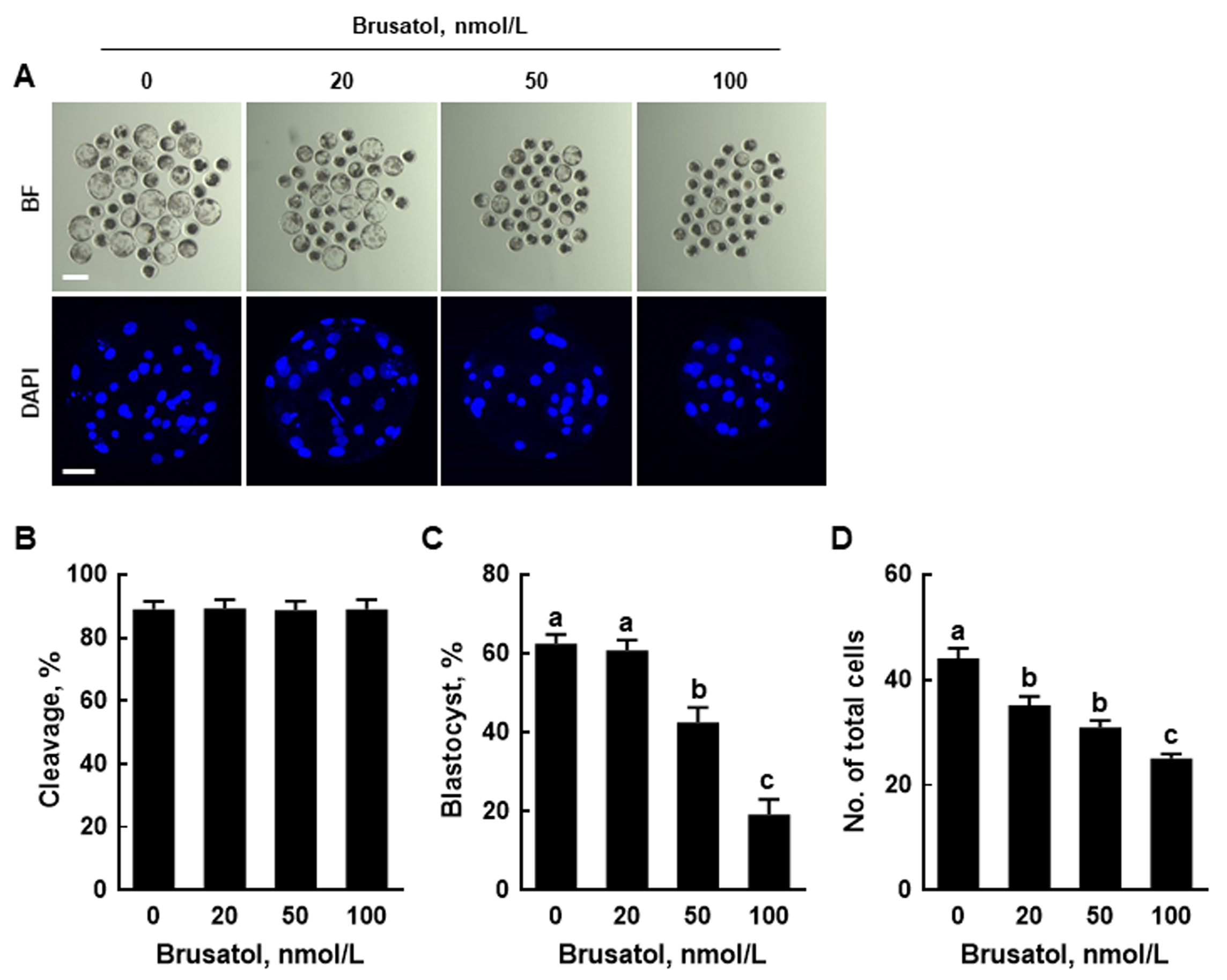

Supplement: Supplementary file 10 — Additional file 10:Fig. S2. Effects of Brusatol on Developmental Competence of Porcine PA Embryos. A Representative bright-field (upper, scale bar = 200 µm) and nuclear-stained (lower, scale bar = 50 µm) images of blastocysts cultured with or without varying concentrations of brusatol. B-D Quantification of the cleavage rate, blastocyst formation rate, and total cell number in the indicated groups (0; n = 181, 20; n = 182, 50; n = 181, 100; n = 181). The data are derived from five independent experiments, and means with similar superscripts do not differ (P > 0.05). [file 40104_2023_947_MOESM10_ESM.tif]
